# A Dual-Laser Sensor Based on Off-Axis Integrated Cavity Output Spectroscopy and Time-Division Multiplexing Method

**DOI:** 10.3390/s20216192

**Published:** 2020-10-30

**Authors:** Kunyang Wang, Ligang Shao, Jiajin Chen, Guishi Wang, Kun Liu, Tu Tan, Jiaoxu Mei, Weidong Chen, Xiaoming Gao

**Affiliations:** 1 Anhui Institute of Optics and Fine Mechanics, Hefei Institutes of Physical Science, Chinese Academy of Sciences, Anhui 230031, China; hahale@mail.ustc.edu.cn (K.W.); shaolg@mail.ustc.edu.cn (L.S.); jjchen@aiofm.ac.cn (J.C.); gswang@aiofm.ac.cn (G.W.); liukun@aiofm.ac.cn (K.L.); tantu@aiofm.ac.cn (T.T.); jxmei@aiofm.ac.cn (J.M.); 2 Science Island Branch of Graduate School, University of Science and Technology of China, Anhui 230031, China; 3 Laboratoire de Physicochimie de l’Atmosphère, Université du Littoral Côte d’Opale, 189A, Av. Maurice Schumann, 59140 Dunkerque, France; chen@univ-littoral.fr

**Keywords:** OA-ICOS, dual-laser sensor, time-division multiplexing, carbon dioxide, methane

## Abstract

In this article, a compact dual-laser sensor based on an off-axis integrated-cavity output spectroscopy and time-division multiplexing method is reported. A complete dual-channel optical structure is developed and integrated on an optical cavity, which allows two distributed feedback (DFB) lasers operating at wavelengths of 1603 nm and 1651 nm to measure the concentration of CO_2_ and CH_4_, simultaneously. Performances of the dual-laser sensor are experimentally evaluated by using standard air (with a mixture of CO_2_ and CH_4_). The limit of detection (LoD) is 0.271 ppm and 1.743 ppb at a 20 s for CO_2_ and CH_4_, respectively, and the noise equivalent absorption sensitivities are 2.68 × 10^−10^ cm^−1^ Hz^−1/2^ and 3.88 × 10^−10^ cm^−1^ Hz^−1/2^, respectively. Together with a commercial instrument, the dual-laser sensor is used to measure CO_2_ and CH_4_ concentration over 120 h and verify the regular operation of the sensor for the detection of ambient air. Furthermore, a first-order exponential moving average algorithm is implemented as an effective digital filtering method to estimate the gas concentration.

## 1. Introduction

Off-axis integrated cavity output spectroscopy (OA-ICOS) is a powerful spectroscopy technology for gas detection owing to advantages including fast response, high sensitivity and stability [1,2,3,4]. Briefly, OA-ICOS employs a high-finesse optical cavity as an absorption cell that consists of two high-reflectivity mirrors (>99.9%), and continuous-wave laser beams that are coupled into the cavity at an angle and are bounced up to thousands of times to reach a long effective path length, which delivers sensitivity down to parts per trillion and parts per billion [5,6,7]. In traditional OA-ICOS approaches, researchers have generally used single laser sources to measure gas concentration or isotopes [8,9,10,11]. Although these “single-laser-source” OA-ICOS approaches can be applied to most detection experiments, they still cannot achieve the demand of simultaneous multi-gas measurements, especially, for a near-infrared narrow linewidth laser, which is one grand challenge for multi-gas measurement in different wavelength ranges.

In recent years, with the gradual development of OA-ICOS [12,13], there have been two main methods of simultaneous multi-gas detections using dual laser sources: frequency division multiplexing assisted wavelength modulation spectroscopy (FDM-WMS) and time-division multiplexing assisted direct absorption spectroscopy (TDM-DAS). Zheng et al. [14] developed a near-infrared CH_4_ and C_2_H_6_ OA-ICOS sensor system at parts per billion (ppb) concentration levels by using two distributed feedback (DFB) lasers and combining FDM-WMS. This dual-gas sensor architecture shows the advantages of simultaneous C_2_H_2_/CH_4_ detection with a single sensor system of reduced size, without affecting sensitivity and reliability. In addition, there are other advantages of wavelength modulation spectroscopy technology (WMS) of FDM-WMS, such as, harmonic detection, near-zero background, zero-absorption baseline and suppression of the 1/f noise [15,16]. However, compared to direct absorption spectroscopy technology (DAS) of TDM-DAS, the analysis of WMS absorption is more complex [17]. While, DAS technology has an important position in the field of detection for its relatively simpler data interpretation and implementation [18,19]. At the same time, DAS technology is also widely used to investigate the spectroscopic parameters [20,21]. In addition, for “dual-laser-sources” OA-ICOS sensors based on TDM-DAS, with the need for a single cavity and a single photodetector and without a hardware lock-in amplifier, the cost of the sensors can be reduced.

In this paper, we set up a compact dual-laser OA-ICOS sensor combining TDM-DAS. The sensor adopts two narrow linewidth DFB lasers operating at wavelengths of 1603 nm and 1651 nm for simultaneously measuring the concentration of CO_2_ and CH_4_. A complete dual-channel optical structure is integrated in the cavity, which allows the sensor to tolerate a very high degree of mechanical vibration. The sensor performances were evaluated by a standard air (with a mixture of CO_2_ and CH_4_). Together with a commercial instrument, the sensor was used to measure ambient CO_2_ and CH_4_ concentrations over 120 h. Furthermore, a first-order exponential moving average (EMA) was implemented, which provides an effective digital filtering method to estimate the gas concentration.

## 2. Experimental Design and Methods

### 2.1. The Dual-Laser OA-ICOS Sensor Setup

**Figure 1:**
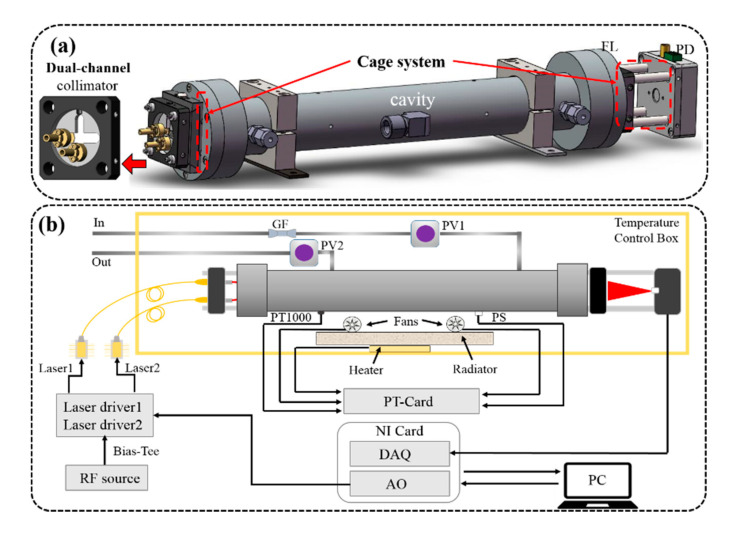
(**a**) A designed cavity including integrated light path and dual-channel incident structure. (**b**) Schematic diagram of the dual-laser off-axis integrated cavity output spectroscopy (OA-ICOS) sensor. PS: pressure sensor, FL: focusing lens, PD: photodetector, GF: gas filter, PV1 and PV2: proportional valve, PT: pressure–temperature.

A compact optical sub-system was designed for coupling the dual laser beam into an optical cavity and delivering the output to a photodetector, as exhibited in Figure 1a. A pair of 1”-diameter spherical mirrors (advanced thin films) with a curvature radius of 1000 mm and a reflectivity R≈ 99.996% were used to form an optical resonator cavity. The two mirrors were placed co-axially on both sides of a stainless steel tube, the distance between the mirrors was 28 cm and the inner tube diameter was 20 mm (volume of the tube, 88 cm^3^).

The outside of the optical cavity contains two optical cage systems (30 mm, Thorlabs, Newton, NJ, USA) by using eight rigid steel rods to mount all optical components along the optical cavity axis. The right system was an output cage system including a focusing lens with a focal length of 10 mm and an InGaAs photodiode (Thorlabs, FGA 10) detecting the light transmitted from the optical cavity. The left system was an input cage system, which allowed the dual-channel lasers to be injected in the optical cavity, simultaneously. The input cage system mainly contains a cage plate (shown as “Dual-channel collimator” in Figure 1a) with a circular-cross structure, which allows the lasers to be placed off-axis. In this way the lasers are coupled “off-axis” into the optical cavity at an angle with respect to the optical axis of the optical cavity. Two adjustable home-made collimators that provide the angle of ±5° tip/tilt along the optical axis were placed on the left and bottom of the cage plate. In addition, the positions of the collimators are not fixed, and they can be placed on any side of the cage plate. Furthermore, the circular-cross structure of the cage plate maximally reduces the physical squeeze and collision between the two collimators, and allows the collimators to be integrated more easily. So, this design is very user-friendly and suitable for the dual-laser OA-ICOS sensor.

Combining the structure of the portable optical sub-system, the dual-laser OA-ICOS sensor designed is depicted in Figure 1b. The home-made laser drivers, which integrated with the temperature controllers and current drivers, were used for driving the lasers. An NI card (National Instruments, NI 6353, Austin, TX, USA), integrated with the analog output (AO) and analog input (AI) functions, was applied as a function generator and data acquisition card. A radio frequency (RF) white noise source (Shenzhen Dakofeng Techno logy Co., Ltd., Shenzhen, China, NF-1000, output frequency range of 5 MHz–1.5 GHz) with a power of −20 dBm was used to broaden the laser linewidth [22,23]. Typically, by broadening the laser linewidth wider than the cavity free spectral range (FSR) by injecting white noise to the laser current, the spurious coupling of the longitudinal cavity modes can be reduced, and the signal-to-noise ratio (SNR) can be increased [24]. A temperature control box was added to provide a stable working temperature for optical detection. The surrounding air in the box was heated by a heating plate, a home-made radiator, and two home-made fans. The temperature and pressure in the box were controlled and stabilized by a home-made PT card (pressure–temperature card), a temperature sensor (Heraeus, M222 PT1000, Hanau, Germany), a pressure sensor (MEAS, US266-000006-005PA, Hampton, VA, USA) and two proportional valves (Clippard, ET-PM-10-4025, Cincinnati, OH, USA).

### 2.2. Selection of CO_2_ and CH_4_ Absorption Lines

**Figure 2:**
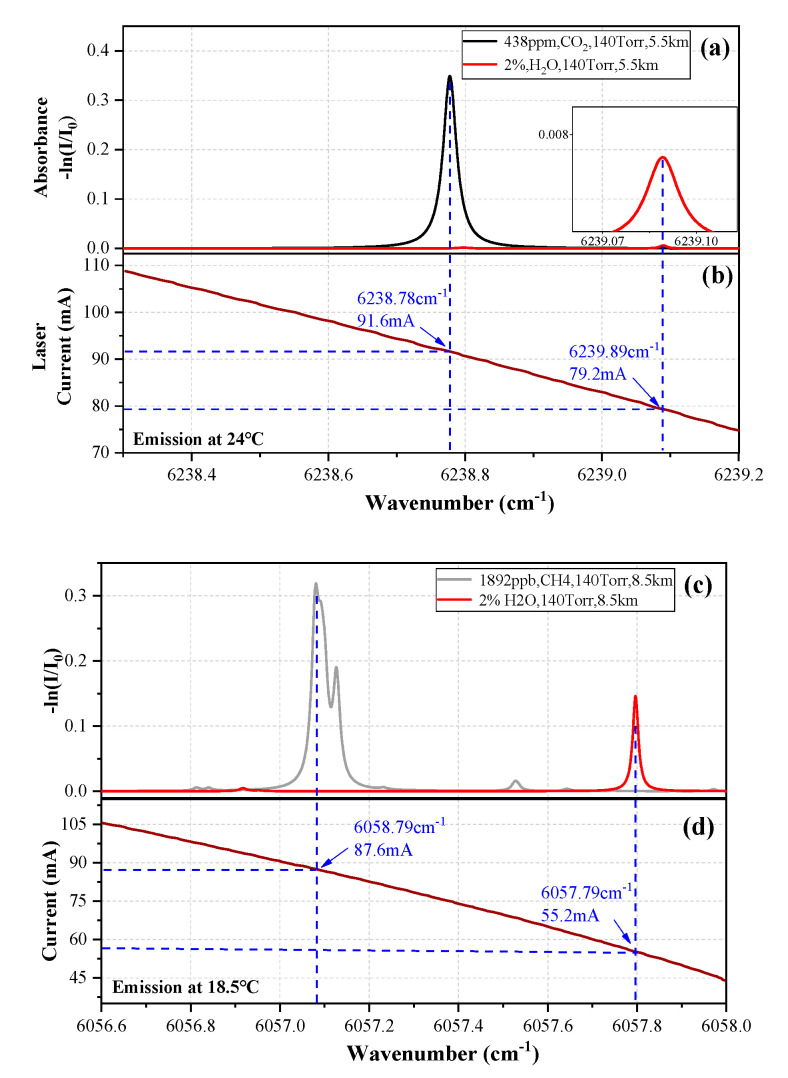
(**a**) HITRAN based absorption spectra of CO_2_ and H_2_O (**b**) Plot of the CO_2_ laser emission wavenumber as a function of the laser drive current. (**c**) HITRAN based absorption spectra of CH_4_ and H_2_O (**d**) Plot of the CH_4_ laser emission wavenumber as a function of the laser drive current.

The 1603.2 nm DFB laser (NEL, NLK1L5EAAA, Oslo, Norway) and the 1651 nm DFB laser (NEL, NLK1U5FAAA) were placed on the “Dual-channel collimator” cage plate and were used to detect the CO_2_ and CH_4_ concentration, respectively. According to the above method, the optical path lengths of the cavity are 5.5 km (@1603 nm, CO_2_) and 8.5 km (@1651 nm, CH_4_), so three transmission spectra of 438.6 ppm CO_2_, 1892.2 ppb CH_4_ and 2% H_2_O in the spectral region from 6238.3 to 6239.2 cm^−1^ and 6056.8 to 6058 cm^−1^, respectively, were simulated using the HITRAN 2016 database. As shown in Figure 2a, the absorption line located at 6238.78 cm^−1^ for high-precision detection of CO_2_ was selected due to its large absorption strength of 1.75 × 10^−23^ cm/molecule at a temperature of 296 K. Furthermore, its absorbance can be up to two orders of magnitude higher than the red line of 2% H_2_O (line strength, 1.23 × 10^−26^ cm/molecule) in the nearby waveband. The CH_4_ absorption lines (three overlapping lines) near 6057.09 cm^−1^, whose full strength is 3.25 × 10^−21^ cm/molecule at a temperature of 296 K, are plotted in Figure 2c. Their distance from the red line of H_2_O (wavenumber, 6057.796 cm^−1^) is 0.705 cm^−1^. At the same time, the linear relationship between the lasers emitting wavenumber and driving current is shown in Figure 2b,d, the current range was decided to be 75−110 mA to scan the CO_2_ line, and the range was decided to be 44−105 mA to simultaneously scan CH_4_ lines (three overlapping lines) and H_2_O lines.

### 2.3. Time-Division Multiplexing Method in the Dual-Laser OA-ICOS Sensor

**Figure 3:**
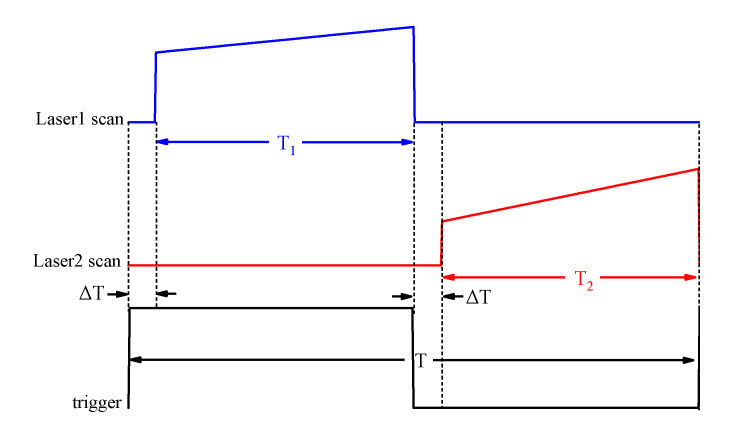
The scanning signal of two lasers and time sequence of the time division multiplexing method. T: periodicity, T_1_: CO_2_ spectral region, T_2_: CH_4_ spectral region, ΔT: dark current of the detector.

**Figure 4:**
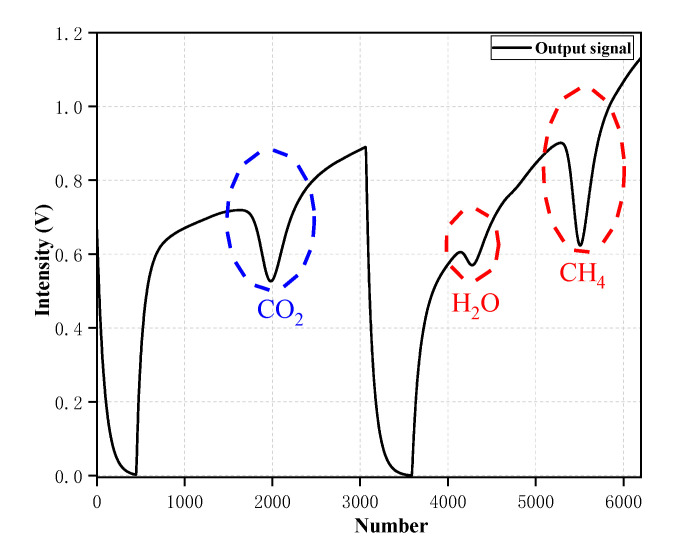
Plot the actual CO_2_ and CH_4_ output signals of the photodetector based on the time division multiplexing method, where bule dash circle represents the CO_2_ absorption line, and red dash circle represents the CH_4_ absorption line.

In order to achieve the purpose of simultaneous detection, a time-division multiplexing method was proposed. Figure 3 shows the scanning signal of two lasers and time sequence of the system. On a scan periodicity T, T_1_ is the scanning range of the laser1 and T_2_ is the scanning range of the laser2, which cover the absorption line of CO_2_ and the absorption line of CH_4_, respectively. “T_2_+ΔT” is a direct current output of laser1 and “T_1_+ΔT” is a direct current output of laser2, which ensure each scanning range of the lasers was staggered without interference. In this way, the laser beams can be simultaneously coupled into the cavity and acquired by the photodetector. Figure 4 depicts the time-division multiplexing method which is used to detect the actual output signals of the photodetector. As shown in Figure 4, the method can be perfectly applied to the dual-laser OA-ICOS sensor without the problem of superposition of laser intensity.

### 2.4. Performance Optimization Using Exponential Moving Average

For the dual-laser OA-ICOS sensor, it is highly desirable to be able to perform real-time concentration measurements with high precision, while the main factors limiting measurement precision are the presence of system noises. These noises from practical measurements enable researchers to use various digital processing methods [25,26,27,28] to estimate the gas concentration. The exponential moving average (EMA) algorithm [29,30], as an effective digital filtering method, can filter these noises and increase the precision of the OA-ICOS sensor.

The EMA can be defined as a linear transformation of raw concentration to a filtered concentration. Let *x_t_* be the raw concentration at time *t* and *x*′*_t_* be the filtered concentration at the same time, the equation can be written as follow:

(1) $${x^{\prime }}_{t}=\left(1-\frac{2}{N+1}\right){x^{\prime }}_{t-1}+\frac{2}{N+1}x_{t}\;\;(N\geq 1)$$

where *N* is length of concentration samples, when *N* = 1, the EMA is the identity transformation: *x*′*_t_*= *x_t_*. Note that the length of concentration samples *N* is important, because as a filter parameter, it determines the final filtered concentration of the OA-ICOS sensor. For a small *N* value, the precision of the filtered concentration is not high, but the sensor’s response time is shorter to follow real-time variation. Conversely, the filtered concentration is more efficient in removing shot-to-shot real-time noises when a large *N* is used, but the sensor’s response time is longer.

**Figure 5:**
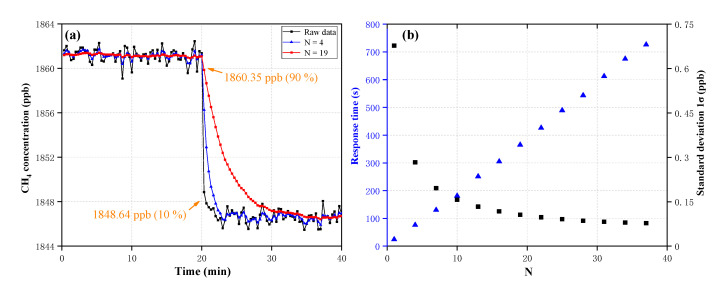
(**a**) Plots of the concentration in 1861.6 ppb and 1847.2 ppb CH_4_ gases, and the two filtering results calculated by the exponential moving average (EMA) algorithm. (**b**) Response time (blue) and standard deviation (black).

Figure 5a shows the concentration of two standard CH_4_ gases (1847.2 ppb and 1861.6 ppb) over a time period of ~40 min, with a measurement interval of 20 s. The concentration inversion method for determining the concentration has been given in Section 3.2. The blue triangle curve and the red circular curve represent the filtered concentration of *N* = 4 and *N* = 19, respectively. Response time is determined by the concentration decreasing from 1860.35 ppb to 1848.64 ppb (orange rectangular area), which is 90% and 10% of the difference between the two concentrations, respectively. Figure 5b shows the relationship between *N* versus the standard deviation of the measurement of 1861.6 ppb CH_4_ gas (black curve) and the sensor’s response time (blue curve). It can be seen that the standard deviation decreases exponentially with increased *N*, and the response time increases linearly with increased N, meanwhile, for the OA-ICOS sensor, a value of *N* = 10 (a quarter of the standard deviation of the raw concentration) was chosen as a compromise between the fast temporal response and the high filtering efficiency. Note that the different concentrations’ standard deviations and the different response time, affect the selection of *N*, thus the experimental conditions were needed to analyze specifically.

## 3. Results and Discussion

### 3.1. The Stability of Temperature and Pressure

**Figure 6:**
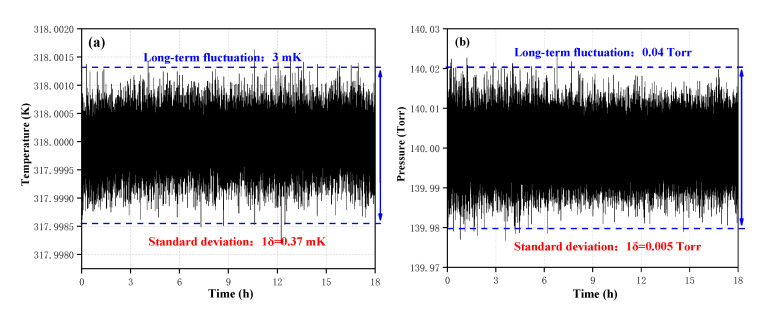
Measurements of temperature (**a**) and pressure (**b**) over an 18 h period of continuous measurement.

In the practical atmospheric measurement, ambient air has a large temperature difference between day and night, such broad temperature changes have a significant impact on the line-strength of the absorption line. In addition, pressure changes also have a significant impact on the linewidth of the absorption line. Therefore, for high precision optical detection, it is necessary to ensure a constant temperature and constant pressure. The data for the target temperature in the cavity stabilized at 318 K (45 °C) and the target pressure stabilized at 140 Torr; recorded in the room-temperature laboratory, with a measurement interval of 1s. Over an 18 h period of continuous measurement, without additional calibration, the standard deviation 1σ was 0.37 mK, the long-term fluctuation value of the temperature was 3 mK and plotted in Figure 6a. The gas-flow of 100 sccm, controlled by a precision gas flow-meter (Beijng Sevenstar Flow Co., Ltd. D07-19B, Beijing, China), at the gas-inlet of the sensor by the gas flow-meter to ensure stable gas. As shown in Figure 6b, the standard deviation 1δ was 0.005 Torr, and the long-term fluctuation value of the pressure was 0.04 Torr.

### 3.2. Calibration

The dual-laser OA-ICOS sensor was used for the quantitative detections of CO_2_ and CH_4_ concentrations. Twelve CO_2_ samples with a concentration range of 50−1200 ppm were prepared by a gas mixing system (Environics. Inc, Series 4000) via diluting a standard 2000 ppm CO_2_ with pure nitrogen N_2_. By using the same gas mixing system, thirteen CH_4_ samples with a concentration range of 800−2300 ppb were prepared via diluting a standard 2300 ppb CH_4_ with pure nitrogen N_2_. In order to ensure that the pressure and temperature in the calibration are consistent with the pressure and temperature in the practical atmospheric measurement, the target pressure and target temperature in the optical cavity were also controlled at 140 Torr, and 45 °C, respectively. The X-axis is the peak height from the spectral fit and the Y-axis is the expected concentration.

**Figure 7:**
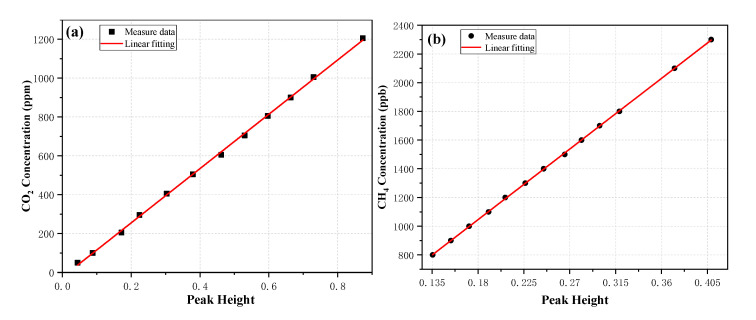
Correlation between CO_2_ (**a**) and CH_4_ (**b**) concentration and peak height (*I*_0_*/I* − *1*) of absorption spectra (from spectral fit).

Figure 7a shows the evolution of the CO_2_ concentration with the absorption term (*I*_0_/*I* − *1*), where *I*_0_ is the incident laser intensity, and *I* is the output intensity of the cavity. A linear fitting equation was obtained with a correlation coefficient R^2^ = 0.99939:

(2) $$\chi_{{\mathrm{CO}}_{2}}=1396.86098-24.45397\times \left(\frac{I_{0}}{I}-1\right)$$

Figure 7b shows the evolution of the CH_4_ concentration with the absorption term (*I*_0_*/I* − *1*). A linear fitting equation was obtained with a correlation coefficient R^2^ = 0.99989:

(3) $$\chi_{{\mathrm{CH}}_{4}}=61.41737+5468.19325\times \left(\frac{I_{0}}{I}-1\right)$$

### 3.3. Long-Term Stability

**Figure 8:**
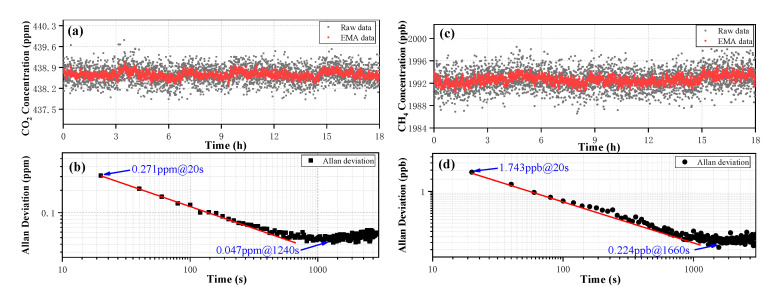
(**a**) The long-term concentration measurement of CO_2_ and its EMA algorithm result. (**b**) Allan deviation analysis of the CO_2_ data. (**c**) The long-term concentration measurement of CH_4_ and its EMA algorithm result. (**d**) Allan deviation analysis of the CH_4_ data.

With a measurement interval of ~20s, spectra of the synthesized air sample (with a mixture of 438.6 ppm CO_2_ and 1992.2 ppb CH_4_) were recorded for ~18 h to evaluate the measurement precision linked to the fluctuation in the measured concentration. The long-term fluctuation range in the CO_2_ concentration was 437.84–439.49 ppm during the whole measurement procedure. In order to further improve the measurement precision, the data of the concentration was processed in real-time using the EMA algorithm. The long-term fluctuation range of CO_2_-EMA reached 438.35–438.97 ppm, plotted in Figure 8a. Similarly, the long-term fluctuation range in the CH_4_ concentration was 1986.89–1998.08 ppb, and down to 1990.72–1994.58 ppb by using the EMA algorithm, as plotted in Figure 8c. Furthermore, the raw long-term 1δ of CO_2_ and CH_4_ was 0.278 ppm and 1.794 ppb and the EMA long-term 1δ of CO_2_ and CH_4_ was 0.107 ppm and 0.733 ppb, as shown in Table 1.

The stability and the detection limits of the dual-laser OA-ICOS sensor can conveniently be Allan deviation. The Allan figure can provide information about an optimum averaging time and a limit of detection (LoD) [31]. Thus, Allan deviation analysis was implemented on the raw data of CO_2_ and CH_4_ to characterize the stability, as plotted in a log–log scale versus the averaging time in Figure 8b,d, respectively. The LoD of 0.271 ppm for a 20 s averaging time, and a minimum LoD of 0.047 ppm for an optimum averaging time of 1240 s were obtained based on the analysis of the CO_2_ concentration. Similarly, For the CH_4_ concentration, a LoD of 1.743 ppb for a 20s averaging time and a minimum LoD of 0.224 ppb for an optimum averaging time of 1660 s were obtained. Using the method described by Baer et al. [32], the detection bandwidths of CO_2_ and CH_4_ were 2.65 Hz (fcavity_CO_2_ =8.5 kHz, 3200 sweeps averaged) and 1.75 Hz (fcavity_CH_4_ =5.6 kHz, 3200 sweeps averaged), respectively. Noise equivalent absorption sensitivities (NEAS) of the CO_2_ and CH_4_ for a 20 s averaging time were 2.68 × 10^−10^ cm^−1^ Hz^−1/2^ and 3.88 × 10^−10^ cm^−1^ Hz^−1/2^, respectively.

### 3.4. Detection of Ambient Air with the Dual-Laser OA-ICOS Sensor

**Figure 9:**
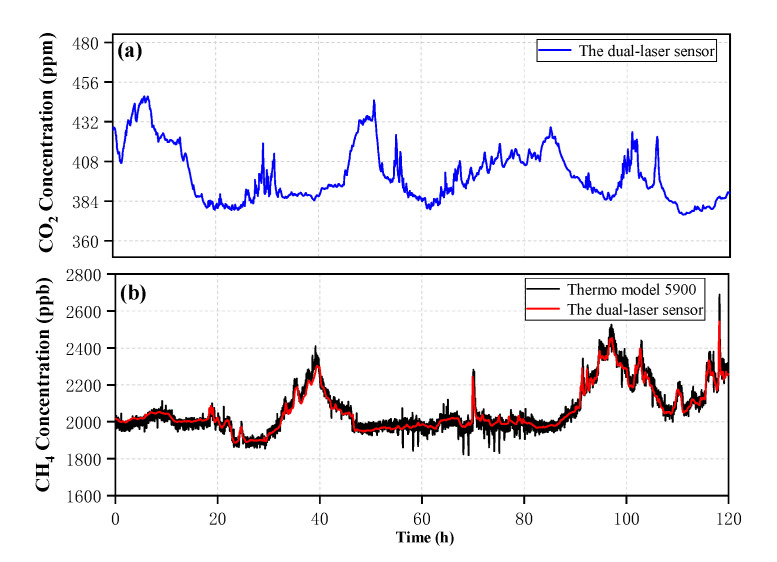
(**a**) The ambient air measurement of carbon dioxide over 120 h. (**b**) The ambient air measurement of methane over 120 h.

Figure 9 depicts the performance of the dual-laser OA-ICOS sensor that is demonstrated with gas concentration measurements in ambient air. A commercial instrument (Thermo Scientific model 5900) was applied to ambient air measurements as well for side-by-side intercomparison. This instrument mainly uses meteorological chromatography technology to detect methane and non-methane total hydrocarbons, so unfortunately, it is impossible to compare the results of carbon dioxide detection. The entire measurements were performed over a time period of ~120 h with a measurement interval of ~120 s. Furthermore, these two instruments were used in a designated meteorological station located in Zibo City, Shandong Province, China. The black solid line represents the CH4 concentration result detected by the commercial instrument, and the red solid line represents the CH4 concentration detected by the dual-laser sensor. The measurement trends of the two instruments are well consistent for the 120 h measurement time. It is proved that the dual-laser sensor has a practical engineering value for the ambient air measurement of gas concentration.

## 4. Conclusions

A compact dual-laser sensor based on OA-ICOS and the time-division multiplexing method was developed in this article. A complete dual-channel optical structure was developed and integrated in the cavity, which allows two DFB lasers operating at wavelengths of 1603 nm and 1651 nm to measure the concentration of CO_2_ and CH_4_, simultaneously. The sensor performances were evaluated by a standard air (with a mixture of CO_2_ and CH_4_). A limit of detection (LoD) was 0.271 ppm and 1.743 ppb at a 20 s, for CO_2_ and CH_4_, respectively, and the noise equivalent absorption sensitivity was 2.68 × 10^−10^ cm^−1^ Hz^−1/2^ and 3.88 × 10^−10^ cm^−1^ Hz^−1/2^, respectively. Together with a commercial instrument, the sensor was used to measure the CO_2_ and CH_4_ concentration over 120 h and verify the regular operation of the sensor for the detection of ambient air. Furthermore, the first-order exponential moving average algorithm is implemented to provide an effective digital filtering method to estimate the gas concentration.

## Figures and Tables

**Table 1:** The long-term 1δ of CO_2_ and CH_4._

	Raw Long-Term 1δ	EMA Long-Term 1δ
CO_2_	0.278 ppm	0.107 ppm
CH_4_	1.794 ppb	0.733 ppb

## References

[1] Sun K., Wang S.K., Sur R., Chao X., Jeffries J.B., Hanson R.K. (2014). Time-resolved in situ detection of CO in a shock tube using cavity-enhanced absorption spectroscopy with a quantum-cascade laser near 4.6 mu m. Opt. Express.

[2] Nadeem F., Mandon J., Cristescu S.M., Harren F.J.M. (2018). Intensity enhancement in off-axis integrated cavity output spectroscopy. Appl. Opt..

[3] Shen G., Chao X., Sun K. (2018). Modeling the optical field in off-axis integrated-cavity-output spectroscopy using the decentered Gaussian beam model. Appl. Opt..

[4] Chao X., Shen G., Sun K., Wang Z., Meng Q., Wang S., Hanson R.K. (2019). Cavity-enhanced absorption spectroscopy for shocktubes: Design and optimization. Proc. Combust. Inst..

[5] Rao G.N., Karpf A. (2011). Extremely sensitive detection of NO₂ employing off-axis integrated cavity output spectroscopy coupled with multiple-line integrated absorption spectroscopy. Appl. Opt..

[6] Karpf A., Rao G.N. (2015). Real-time trace gas sensor using a multimode diode laser and multiple-line integrated cavity enhanced absorption spectroscopy. Appl. Opt..

[7] Centeno R., Mandon J., Cristescu S.M., Harren F.J.M. (2014). Sensitivity enhancement in off-axis integrated cavity output spectroscopy. Opt. Express.

[8] Kasyutich V.L., Martin P.A., Holdsworth R.J. (2006). An off-axis cavity-enhanced absorption spectrometer at 1605 nm for the (CO_2_)-C-12/(CO_2_)-C-13 measurement. Appl. Phys. B Lasers Opt..

[9] Moyer E.J., Sayres D.S., Engel G.S., Clair J.M.S., Keutsch F.N., Allen N.T., Kroll J.H., Anderson J.G. (2008). Design considerations in high-sensitivity off-axis integrated cavity output spectroscopy. Appl. Phys. B Lasers Opt..

[10] Kireev S.V., Kondrashov A.A., Shnyrev S.L. (2018). Improving the accuracy and sensitivity of(13)C online detection in expiratory air using the TDLAS method in the spectral range of 4860-4880 cm(−1). Laser Phys. Lett..

[11] Alquaity A.B.S., Utsav K.C., Popov A., Farooq A. (2017). Detection of shock-heated hydrogen peroxide—(H_2_O_2_) by off-axis cavity-enhanced absorption spectroscopy (OA-CEAS). Appl. Phys. B Lasers Opt..

[12] Zheng K., Zheng C., Li J., Ma N., Liu Z., Zhang Y., Wang Y., Tittel F.K. (2020). Near-infrared methane sensor system using off-axis integrated cavity output spectroscopy with novel dual-input dual-output coupling scheme for mode noise suppression. Sens. Actuators B Chem..

[13] Berman E.S.F., Fladeland M., Liem J., Kolyer R., Gupta M. (2012). Greenhouse gas analyzer for measurements of carbon dioxide, methane, and water vapor aboard an unmanned aerial vehicle. Sens. Actuators B Chem..

[14] Zheng K., Zheng C., Yao D., Hu L., Liu Z., Li J., Zhang Y., Wang Y., Tittel F.K. (2019). A near-infrared C2H2/CH4 dual-gas sensor system combining off-axis integrated-cavity output spectroscopy and frequency-division-multiplexing-based wavelength modulation spectroscopy. Analyst.

[15] Yang C., Mei L., Deng H., Xu Z., Chen B., Kan R. (2019). Wavelength modulation spectroscopy by employing the first harmonic phase angle method. Opt. Express.

[16] Goldenstein C.S., Spearrin R.M., Jeffries J.B., Hanson R.K. (2017). Infrared laser-absorption sensing for combustion gases. Prog. Energy Combust. Sci..

[17] Sun K., Chao X., Sur R., Goldenstein C.S., Jeffries J.B., Hanson R.K. (2013). Analysis of calibration-free wavelength-scanned wavelength modulation spectroscopy for practical gas sensing using tunable diode lasers. Meas. Sci. Technol..

[18] Goldenstein C.S., Jeffries J.B., Hanson R.K. (2013). Diode laser measurements of linestrength and temperature-dependent lineshape parameters of H_2_O-, CO_2_-, and N_2_-perturbed H_2_O transitions near 2474 and 2482 nm. J. Quant. Spectrosc. Radiat. Transf..

[19] Buldyreva J., Margules L., Motiyenko R.A., Rohart F. (2013). Speed dependence of CH_3_ Cl-35-O-2 line-broadening parameters probed on rotational transitions: Measurements and semi-classical calculations. J. Quant. Spectrosc. Radiat. Transf..

[20] Pogány A., Klein A., Ebert V. (2015). Measurement of water vapor line strengths in the 1.4–2.7 µm range by tunable diode laser absorption spectroscopy. J. Quant. Spectrosc. Radiat. Transf..

[21] Cai T., Wang G., Cao Z., Zhang W., Gao X. (2014). Sensor for headspace pressure and H2O concentration measurements in closed vials by tunable diode laser absorption spectroscopy. Opt. Lasers Eng..

[22] Wang J., Tian X., Dong Y., Zhu G., Chen J., Tan T., Liu K., Chen W., Gao X. (2019). Enhancing off-axis integrated cavity output spectroscopy (OA-ICOS) with radio frequency white noise for gas sensing. Opt. Express.

[23] Wang J., Tian X., Dong Y., Chen J., Tan T., Zhu G., Chen W., Gao X. (2019). High-sensitivity off-axis integrated cavity output spectroscopy implementing wavelength modulation and white noise perturbation. Opt. Lett..

[24] Engel G.S., Moyer E.J., Keutsch F.N., Anderson J.G. (2003). Innovations in Cavity Enhanced Laser Absorption Spectroscopy: Using in situ Measurements to Probe the Mechanisms Driving Climate Change. https://www.researchgate.net/profile/E_Moyer/publication/237635995_Innovations_in_Cavity_Enhanced_Laser_Absorption_Spectroscopy_Using_in_situ_Measurements_to_Probe_the_Mechanisms_Driving_Climate_Change/links/5417c5cb0cf2f48c74a4112a.pdf.

[25] Kireev S.V., Kondrashov A.A., Shnyrev S.L., Frolov N.V. (2018). Kalman’s method to improve accuracy of online (CO2)-C-13-O-16 measurement in the exhaled human breath using tunable diode laser absorption spectroscopy. Laser Phys. Lett..

[26] Wu T., Chen W., Fertein E., Masselin P., Gao X., Zhang W., Wang Y., Koeth J., Brueckner D., He X. (2014). Measurement of the D/H, O-18/O-16, and O-17/O-16 Isotope Ratios in Water by Laser Absorption Spectroscopy at 2.73 mu m. Sensors.

[27] Schiller C.L., Bozem H., Gurk C., Parchatka U., Königstedt R., Harris G.W., Lelieveld J., Fischer H. (2008). Applications of quantum cascade lasers for sensitive trace gas measurements of CO, CH_4_, N_2_O and HCHO. Appl. Phys. B.

[28] Xie Q., Li J., Gao X., Jia J. (2010). Fourier domain local narrow-band signal extraction algorithm and its application to real-time infrared gas detection. Sens. Actuator B Chem..

[29] Grebenkov D.S., Serror J. (2014). Following a trend with an exponential moving average: Analytical results for a Gaussian model. Phys. A Stat. Mech. Appl..

[30] Tsokos C.P. (2010). K-th Moving, Weighted and Exponential Moving Average for Time Series Forecasting Models. Eur. J. Pure Appl. Math..

[31] Werle P., Mücke R., Slemr F. (1993). The limits of signal averaging in atmospheric trace-gas monitoring by tunable diode-laser absorption spectroscopy (TDLAS). Appl. Phys. B.

[32] Baer D.S., Paul J.B., Gupta J.B., O’Keefe A. (2002). Sensitive absorption measurements in the near-infrared region using off-axis integrated-cavity-output spectroscopy. Appl. Phys. B Lasers Opt..

